# Herniarin, Dimethylfraxetin and Extracts from *Tagetes lucida*, in Psychosis Secondary to Ketamine and Its Interaction with Haloperidol

**DOI:** 10.3390/plants11202789

**Published:** 2022-10-21

**Authors:** Sandra Liliana Porras-Dávila, Enrique Jiménez-Ferrer, Rubén Román Ramos, Manasés González-Cortazar, Julio César Almanza-Pérez, Maribel Herrera-Ruiz

**Affiliations:** 1 Centro de Investigación Biomédica del Sur, Instituto Mexicano Del Seguro Social, Argentina No. 1, Col. Centro, Xochitepec 62790, Morelos, Mexico; 2 Doctorado en Ciencias Biológicas y de la Salud, División de Ciencias Biológicas y de la Salud, Universidad Autónoma Metropolitana (UAM), Ciudad de México 04960, Mexico; 3 Departamento de Ciencias de la Salud, División de Ciencias Biológicas y de la Salud, Universidad Autónoma Metropolitana-Iztapalapa, Av. Ferrocarril San Rafael Atlixco 186, Leyes de Reforma 1era Secc., Ciudad de México 09310, Mexico

**Keywords:** *Tagetes lucida*, psychosis, herniarin, dimethylfraxetin, ketamine, haloperidol

## Abstract

*Tagetes lucida* Cav., is a medicinal plant used in Mexico to alleviate different disorders related to alterations of the central nervous system, such as behaviors associated with psychosis. The present work evaluated the effect of different extracts separated from this plant, TlHex, TlEA, TlMet, and TlAq, and of two isolated coumarins, herniarin (HN) and dimethylfraxetin (DF), on haloperidol-induced catalepsy (HAL), and psychotic behaviors provoked with a glutamatergic antagonist, ketamine (KET) on ICR mice. The extracts TlEA, TlAq, and the isolated compounds HN and DF, induced an increment of the cataleptic effect of HAL. Schizophrenia-like symptoms caused by KET were analyzed through the behavior of the animals in the open field (OFT), forced swimming (FST), passive avoidance test (PAT), and social interaction test (SIT). Treatments derived from *T. lucida* could interact with this substance in all tests except for FST, in which only TlMet blocks its activity. Mainly, TlEA, TlAq, HN, and DF, blocked the effects of KET on stereotyped behavior, hyperlocomotion, cognitive impairment, and detriment in the social interaction of rodents. *T. lucida* interacted with dopaminergic and glutamatergic systems.

## 1. Introduction

There is a wide variety of mental disorders, each with distinct manifestations, characterized by a combination of disturbances in thinking, perception, emotions, behavior, and relationships with others. These include psychosis, mainly schizophrenia [1], a psychiatric illness of genetic and environmental origin characterized by a variable set of classic symptoms of psychosis, the positive symptoms, which involve hallucinations and delusions. The negative symptoms include flat affect, alogia, anhedonia, social deficits, and cognitive symptoms, which reflect deficits in attention, memory, perception, and thinking [2,3]. This disease affects more than 21 million people worldwide. It is more common in men (12 million) than in women (9 million). Moreover, men generally develop schizophrenia at a younger age [1].

Despite advances in understanding the biological basis of behavior, the precise neurobiological mechanisms involved in schizophrenia remain largely unknown, so there are several hypotheses about the etiology of the development of this complex and multifactorial disease [4]. The dopaminergic hypothesis holds that the symptoms of this disease are due to an excess of dopamine or elevated sensitivity to this neurotransmitter system [5]. Furthermore, it postulates that hyperactivity of mesolimbic dopaminergic pathways and alterations of D1 and D2 family receptors and the pre-synaptic space are decisive for the clinical expression of psychosis, with the appearance of positive, negative, affective, and cognitive symptomatology [6].

On the other hand, the glutaminergic system is involved in psychosis symptoms. Therefore, there could be a decrease in the signaling of this pathway at the level of the ionotropic-NMDA receptors. Furthermore, the blockade of these NMDA receptors generates the positive, negative, cognitive, and affective symptoms typical of schizophrenia. Thus, the NMDA hypofunction may make the dopaminergic system more sensitive to psychosocial stress, and it has been argued that dopamine dysregulation in this disorder may even be secondary to glutamatergic impairment and that the prefrontal cortex and hippocampus are potential sites of regulation of dopaminergic neurons via glutamatergic projections to the midbrain [5].

Therapeutic management of this disease is based on the administration of antipsychotic drugs, which have a common (although not unique) mechanism of action: the anti-dopaminergic effect, since they act antagonistically on D2 receptors. These are classified into typical antipsychotics, such as haloperidol and atypical antipsychotics, such as olanzapine [7]. These are effective for negative (atypical) and positive (typical and atypical) symptoms, which results in a benefit for patients with schizophrenia, but which, in turn, as a consequence of prolonged treatment, leads to significant acute and chronic adverse side effects [8].

Therefore, it is necessary to research new therapeutic alternatives that are more secure and effective, as well as valuable and appropriate animal models for the treatment of schizophrenia; for this reason, several plants that have been essential instruments of traditional medicine in different pathologies are now being used as new alternatives.

*Tagetes lucida* Cav., is a plant native to Mexico commonly known in traditional Mexican medicine as “pericón”, among others. It belongs to the Compositae/-Asteraceae family, and its most frequent medicinal use is for digestive disorders. Moreover, for pain in general, inflammation problems, insomnia, anxiety, and depression [9]. It is also widely used to treat ailments such as “nerves” and “fright” for the “CRAZY” as it relieves the insane [10]. Among the pharmacological properties of *Tagetes lucida* are different studies that indicate its anti-inflammatory and antioxidant activity attributed to some of its compounds, such as coumarins, flavonoids, and essential oil [11,12]. Recent studies evaluated the anti-inflammatory activity of hexane and acetone extract of the species in a model of ear edema induced with TPA finding an inhibition of inflammation of 92.7 and 75.9% and from which five coumarins were isolated, among which found HN and DF [13]. Likewise, El-Newary et al. [14] reported the antioxidant effect of the species by modulating biomarkers of oxidative stress, such as malondialdehyde and activities of catalase, superoxide dismutase, glutathione reductase, and glutathione peroxidase in a CCl_4_-induced hepatotoxicity model. It has also been shown to have CNS effects by its antidepressant properties mediated by 5-HT(1A) and 5-HT(2A) receptors in the forced swimming test in rats [15], anxiolytic and sedative effects in experimental models in mice such as open field, exploration cylinder, hole plate, maze plus and potentiation of barbiturate-induced hypnosis [16] and analgesic properties by involving the participation of receptors like opioids, benzodiazepines, and Serotonin 1A receptor 5-HT, as well as nitric oxide.

Due to the ethnomedicinal use of the species, several toxicity studies have been conducted in mice and rats, demonstrating its safety at high doses. Diaz-Samayoa and Brenda. [17] reported that the aqueous extract (1–5 g/kg), administered orally to rats, did not induce signs of gastric toxicity or digestive hemorrhage, and administered orally to mice in the acute toxicity test, showed no toxic effect. El-Newary et al. [14] evaluated toxicity by the Bruce method at 0.5, 1, 1, 1, 2, 3, 4, and 5 g/kg, reporting that the species was safe at the maximum dose. Likewise, Guadarrama-Cruz et al. [18] reported that the aqueous extract of *T. lucida* shows no signs of toxicity or mortality in acute toxicity tests, so this extract provides an introductory level of safety when combined with the lack of severe side effects reported in its use in traditional medicine.

In addition, there are previous reports of the phytochemical compounds produced by *Tagetes lucida* Cav., which include flavonoids, tannins, essential oils and coumarins, which could have beneficial effects on CNS [9].

HN, or 7-methoxycoumarin, is one of the more common coumarins, occurring in several plant families, such as Caryophyllaceae, Gramineae, Labiatae, Leguminosae, Moraceae, Rosaceae, Rutaceae, Solanaceae, and Compositae. Furthermore, it has antifungal, antibacterial, antifungal [19], antigenotoxic [20], radical scavenging, antinociceptive, cytotoxic, antitumoral [21], anxiolytic, antidepressant [22] and also was found as nootropic [23]. On the other hand, DF or 6,7,8-trimethoxycoumarin is a coumarin obtained from species such as *Gomortega keule*. This compound is antioxidative [24], anxiolytic, and sedative-like activities, the last involving serotonergic and GABAergic neurotransmission [16]. These compounds were previously isolated and reported for the species *T. lucida* [13].

This study aimed to assess the effect of extracts and two compounds isolated from *Tagetes lucida* Cav., in mice exposed to potentiation of haloperidol (dopaminergic system) -induced catalepsy using the bar test. Moreover, on symptoms similar to schizophrenia induced with the acute administration of ketamine (glutamatergic system), in this last part, the effects were evaluated by employing behavioral tests, including the passive avoidance test for cognitive deterioration; open field test for positive symptoms; the forced swimming and social interaction test for negative symptoms.

## 2. Results

### 2.1. Quantification and Chemical Identification of Dimethylfraxetine (DF) and Herniarin (HN)

**Figure 1:**
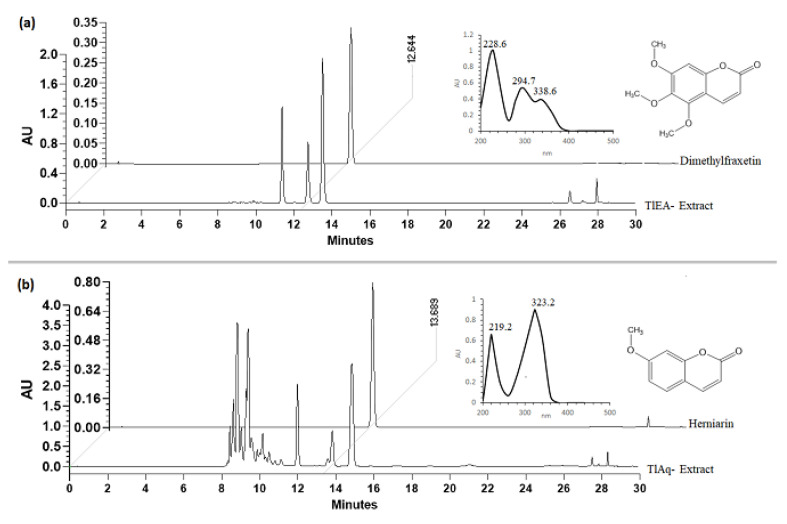
HPLC chromatograms show the chemical profile. AU, absorbance units; (**a**) RT of the TlEA, UV spectrum, and chemical structure of dimethylfraxetin (DF); (**b**) RT of the TlEA, UV spectrum, and chemical structure of herniarin (HN).

HPLC analysis (Figure 1) allowed us to obtain the concentration of DF in the ethyl acetate extract (TlEA), which was 253 mg g^−1^ of extract, and HN in the aqueous extract (TlAq) was 204 mg g^−1^ of extract. The identification of DF and HN was made by comparison with the isolated compounds in the retention times (RT) and UV absorption spectra. The HPLC chromatogram (Figure 1a) of the TlEA extract showed the presence of DF, with an RT of 12.64 min and a UV spectrum with lmax = 228.6, 294.7, and 338.6 nm, and the TlAq extract showed HN with an RT of 13.68 min, and a UV spectrum with lmax = 219.2 and 323.2 nm, characteristic of coumarin-like compounds (Figure 1b).

### 2.2. Catalepsy Induced by HAL Catalepsy Induced by HAL of Four Extracts (TlHex, TlEA, TlMet and TlAq) and Two Compounds (HN and DF)

**Figure 2:**
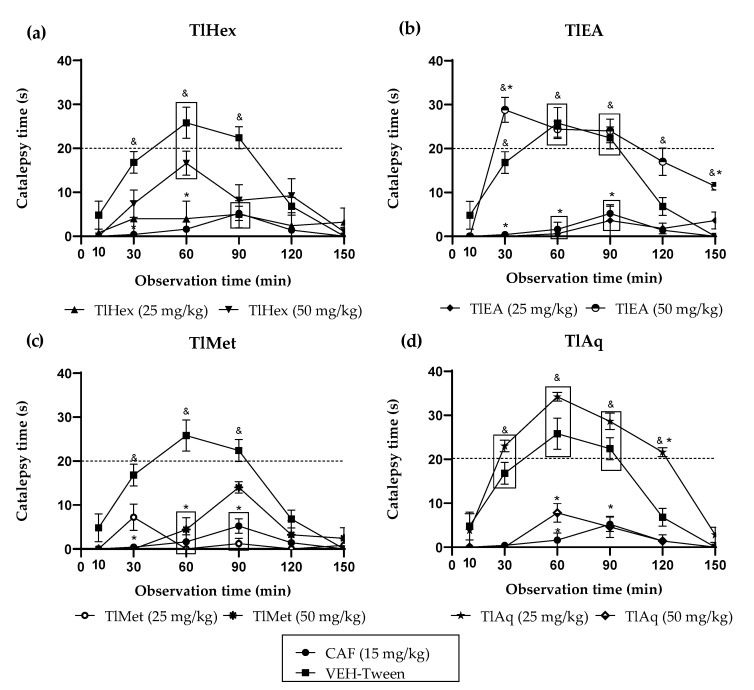
Effect of *T. lucida* extracts on cataleptic state induced with haloperidol (HAL) 0.5 mg/kg i.p., in Bar test (BT) in mice: (**a**) TlHex; (**b**) TlEA; (**c**) TlMet; (**d**) TlAq. ANOVA followed by Dunnett’s Test ($\bar{x}$ ± S.E.M., n = 5); * *p* < 0.05 in comparison with the VEH-Tween group, ^&^ *p* < 0.05 in comparison with caffeine (CAF).

The results obtained in this test (Figure 2) show that the negative control VEH-Tween presented a tendency to increase the immobility time of the mice on the BT from minute 10, an effect that was progressive as time passed. At minute 30, a significant increase in the immobility time on the bar was already observed concerning the caffeine group (CAF 15 mg/kg, ^&^ *p* < 0.05). Nevertheless, it was not until the 60 min and up to the 90 min that the cataleptic effect (staying on the bar without moving for more than 20 s) was present. These data were significantly different also to CAF, which blocked the cataleptic effect provoked by HAL. TlHex treatment at a dose of 50 mg/kg increases the time of immobile posture of the animal. It is presented as a trend from minute 30, with a maximum peak at minute 60, and this does not represent cataleptic activity. Although the administration of TlEA at 50 mg/kg induced a significant increase with respect to the caffeine group, in the time the mice remained in the immobile posture, showing catalepsy from minute 10 to minute 120, even at minute 150, the mice still had traits of this effect (^&^ *p* < 0.05). It should be noted, for this treatment, that the cataleptic activity in minute 30 and minute 150 presented a significant difference with respect to the group of animals receiving only HAL (* *p* < 0.05), and in minute 30, it occurs with a cataleptic time greater than 20 s enhancing HAL action. The lowest dose of TlEA, 25 mg/kg, could completely block the cataleptic state induced by the D2 agonist, behaving similarly to CAF.

TlAq extract at 25 mg/kg, induces a significant increase compared to CAF (^&^ *p* < 0.05) in bar immobility posture, starting at minute 30, with the presence of catalepsy by minute 60 and up to 90, and by minute 120 in addition to the difference with caffeine there is a statistically significant difference over 20 s with the group that only received HAL (* *p* < 0.05), an effect that declines subsequently; a similar pattern as the negative control group is observed. However, the results presented at a dose of 50 mg/kg block the effect of HAL, at all the times analyzed. Both doses of 50 and 25 mg/kg of the TlMet extract decrease HAL-induced catalepsy, with greater blockade observed at the lower dose.

**Figure 3:**
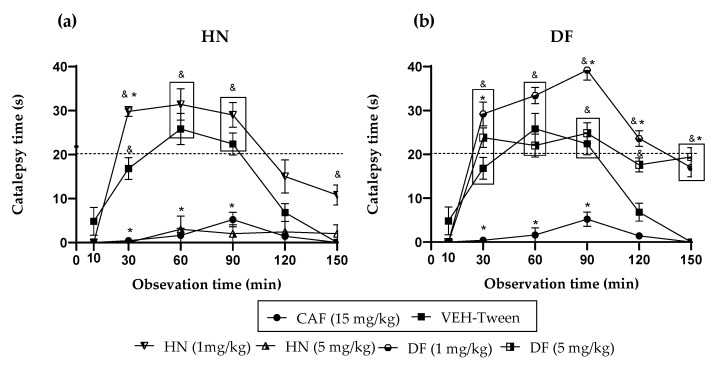
Effect of coumarins isolated from *T. lucida* on the cataleptic state (haloperidol-HAL-0.5 mg/kg i.p.), in Bar test (BT) on mice. (**a**) Herniarin (HN); (**b**) Dimethylfraxetin (DF). ANOVA followed by Dunnett’s Test ($\bar{x}$ ± S.E.M., n = 5); * *p* < 0.05 in comparison with the VEH-Tween group, ^&^ *p* < 0.05 in comparison with caffeine (CAF).

HN and DF, isolated from the active extracts, presented a potentiation of cataleptic effect at doses of 1 mg/kg. The results obtained in this assay (Figure 3) indicate that the group administered with DF, at a dose of 1 mg/kg, presents a positive cataleptic state at minute 30, with a statistically significant difference in comparison with the positive control group (CAF 15 mg/kg) (^&^ *p* < 0.05), this positive effect increased until minute 60 until minute 90 and 120 a cataleptic potentiation is observed with a statistically significant difference with respect to the animals administered only with HAL (* *p* < 0.05). At minute 150, there is a decrease in a catalepsy of fewer than 20 s; however, there is still a statistically significant difference when compared with the positive control group and the VEH-Tween group.

For the dose of DF at 5 mg/kg, a positive cataleptic state can be observed from minute 30 to minute 90 with a significant difference compared with the positive control group CAF (^&^ *p* < 0.05); however, there was no difference with the VEH-Tween group. At minute 120, the cataleptic state decreased progressively, and this effect was maintained until minute 150 so that at these two points, there was a statistically significant difference with the positive control group (^&^ *p* < 0.05) or with the animals administered only with HAL (* *p* < 0.05).

Likewise, HN at 1 mg/kg induced in mice a behavior similar to that of DF at the same dose, with a positive cataleptic state from min 30 and a statistically significant difference with both the positive control group CAF (^&^ *p* < 0.05) and the group of animals only with HAL (* *p* < 0.05) above 20 s, thus generating a potentiation of the cataleptic effect. At minute 90, the cataleptic state increased with a statistically significant difference only with respect to the positive control group (CAF ^&^ *p* < 0.05), and by minutes 120 and 150, it progressively decreased with no statistically significant difference with either group. However, at a dose of 5 mg/kg, a blockage of the HAL effect was observed at all times analyzed.

### 2.3. Interaction of Tagetes lucida Extracts, HN and DF, with KET in Different Behavioral Tests

According to the results obtained from the haloperidol-induced catalepsy assay, the extracts (TlHex, TlEA, and TlMet at 50 mg/kg and TlAq at 25 mg/kg) and the compounds (HN and DF, 1 mg/kg) evaluated in the ketamine-induced psychosis model according to their doses with the highest activity.

#### 2.3.1. Passive Avoidance Test (PAT)

**Figure 4:**
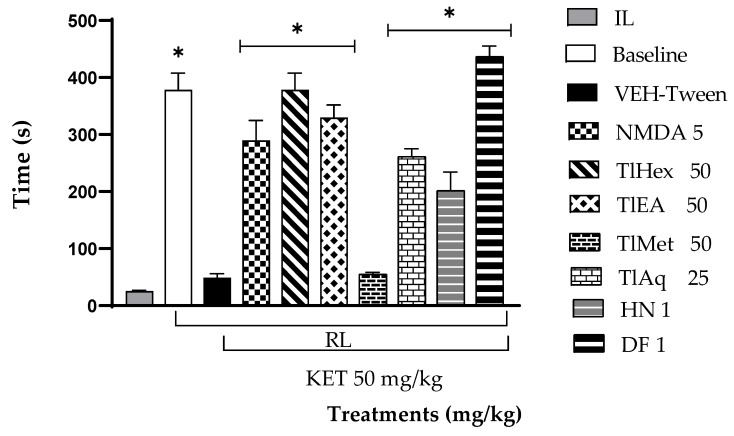
Effect of *T. lucida* extracts, herniarin (HN), and dimethylfraxetin (DF) on acute schizophrenia-like symptoms induced with KET 50 mg/kg i.p., in the Passive avoidance test (PAT) in mice. ANOVA followed by Dunnett’s Test ($\bar{x}$ ± S.E.M., n = 5) with * *p* < 0.05 compared to the VEH-Tween group. RL = retention latency; IL = initial latency.

The effect of *T. lucida* extracts on the cognitive activity of mice administrated with KET (50 mg/kg) and evaluated utilizing the PAT can be observed in Figure 4. The latency time during training, IL (20.85 s average), was the same for all groups, so there was no statistical difference between groups since the animals were in the same conditions at the beginning of the test. In the RL phase, latency time during the test, it can be observed that the VEH-Tween group (negative control) and TlMet 50 mg/kg presented a similar time to the IL. So, this extract did not generate any learning in the rodents; therefore, did not present a statistically significant difference with the damage group (* *p* < 0.05). On the contrary, the baseline group (healthy animals) did not generate any damage and showed normal learning behavior, so they all remembered the aversive stimulus.

The administration of NMDA 5 mg/kg induced an increase in the RL, indicating a protective effect that translates into learning, since they were able to remember the aversive stimulus, and their response time, in this case, was more significant than in the initial stage and the KET alone group (VEH-Tween). Similar behavior was observed in the groups receiving TlHex, and TlEA extracts at 50 mg/kg and TlAq at 25 mg/kg. However, these data differed statistically from the VEH-Tween (* *p* < 0.05).

In the same way, it shows that the RL phase time of DF and HN at 1 mg/kg presented a significant difference in comparison with the VEH-Tween group (*p* < 0.05). Both compounds were able to reverse the damage caused by KET. However, DF presented higher values than the reference drug (NMDA 5 mg/kg) and animals in the baseline group, so a better protective effect translated into learning in rodents can be observed.

#### 2.3.2. Forced Swimming Test (FST)

**Figure 5:**
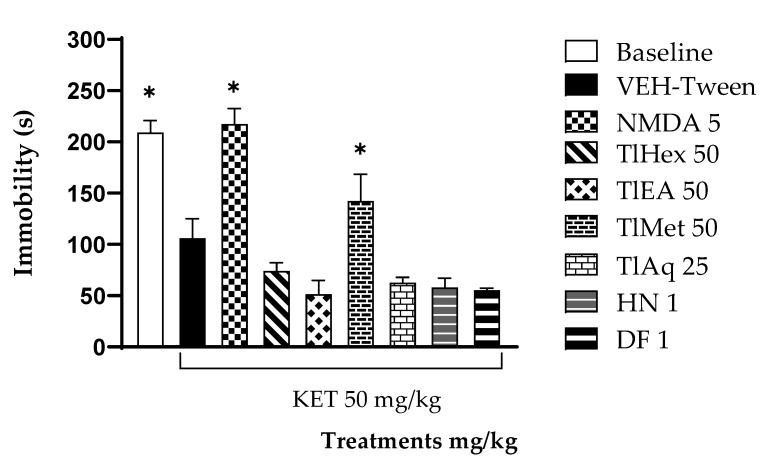
Effect of *T. lucida* extracts, herniarin (HN), and dimethylfraxetin (DF) on acute schizophrenia-like symptoms induced with KET 50 mg/kg i.p., in the Forced Swimming Test (FST) in mice. ANOVA followed by Dunnett’s Test (±S.E.M., n = 5) with * *p* < 0.05 when the comparison is with the VEH-Tween group.

The results obtained in the acute administration of KET were made on animals subjected to FST. Figure 5 indicates that the animals in the baseline group presented a significant difference compared with the negative control group (VEH-Tween). Hence, a depressive effect was present in the animals, which was enhanced by KET increasing the depressive state in the VEH-Tween animals. For of TlHex and TlEA (50 mg/kg), and TlAq (25 mg/kg), it is observed that these induced a significant decrease in the immobility parameter compared with the VEH-Tween group (* *p* < 0.05). As for HN and DF at 1 mg/kg induced a significant decrease in the immobility parameter with respect to the VEH-Tween group, to which no treatment was administered (* *p* < 0.05), these coumarins had similar behavior as KET (VEH-Tween).

#### 2.3.3. Open Field Test (OFT)

In the acute KET induction trial, positive symptoms were evaluated in the OFT (Table 1), indicating that the parameters evaluated in the baseline group presented lower values than the animals that received KET and VEH-Tween, (* *p* < 0.05). While the mice that received the NMDA drug at 5 mg/kg showed similar behavior to the baseline in TC, R, G, TSG, SB, and TSB, all showed statistical differences with VEH-Tween (* *p* < 0.05). In addition, the administration of all the extracts induced a significant decrease in the number of TC compared with the negative control VEH-Tween (* *p* < 0.05). Similarly, the R and G decreased significantly, except for the TlMet group at 50 mg/kg, in which the number of events increased in both parameters, surpassing the negative control without significant difference. Knotted to the results obtained with the different extracts in the Grooming, the animals invested less time in this activity (TSG) with a significant difference in comparison with VEH-Tween (* *p* < 0.05). However, the TlMet group causes only a tendency to decrease that variable but fails to be significantly different from VEH-Tween. Regarding stereotyped behaviors (SB) and time spent in these (TSB), it can be observed that the VEH-Tween damage group presented higher values. TlMet and TlAq extracts were different (* *p* < 0.05), and the TlEA extract did not cause such behaviors, so no value was recorded. It should be noted that TlHex induced a significant decrease in the number of SB to such an extent that only 1 ± 0.63 events were observed; however, the time in which it performed these behaviors (TSB) was similar to that of VEH-Tween (*p* > 0.05).

For DF and HN, it is observed that the administration of the compounds induced a significant decrease in the number of TC, R, and G with a statistically significant difference with the VEH-Tween group (* *p* < 0.05). Furthermore, DF presented TSG values similar to those of the baseline group and the NMDA reference drug. However, although HN presented G values similar to those of the baseline group, it increased the time in which the animals spent grooming per event (TSG), so that in this parameter it did not present a statistically significant difference with the VEH-Tween group (* *p* < 0.05). Stereotyped behaviors (SB) and time spent in these (TSB), it can be observed that both compounds at the dose administered did not cause such behaviors, so no value was recorded, and these results were statistically different from the group of animals administered only with KET (* *p* < 0.05), so there was no psychotic state in the animals, the main positive symptom of patients with schizophrenia.

#### 2.3.4. Social Interaction Test (SIT)

**Figure 6:**
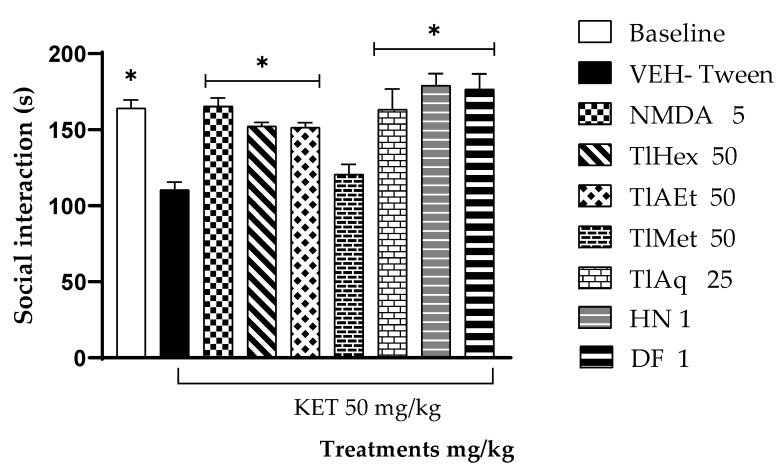
Effect of *T. lucida* extracts, herniarin (HN), and dimethylfraxetin (DF) on acute schizophrenia-like symptoms induced with KET 50 mg/kg in the Social Interaction Test (SIT) in mice. Data show mean ± S.E.M. of 5 animals * *p* < 0.05 indicates statistically significant differences using an ANOVA followed by Dunnett’s Test compared with the VEH-Tween group.

In Figure 6, it can be observed that the animals of the Baseline group, which did not receive ketamine, spent significantly more time smelling a new mouse than those in the group of damage with KET (VEH-Tween * *p* < 0.05). Animals administered with KET and with NMDA at 5 mg/kg, TlHex and TlEA at 50 mg/kg, and TlAq at 25 mg/kg, were able to significantly counteract the effect of KET on sociability, marked as sniffs (* *p* < 0.05). While TlMet does not modify that parameter compared to the group with VEH-Tween. The coumarins DF and HN induced a similar behavior and were able to increase the number of sniffs in comparison with the VEH-Tween group (* *p* < 0.05)

## 3. Discussion

Pharmacological potential for the possible management of psychotic-like symptoms. This plant has reports of ethnomedical uses and pharmacological activities in animal models, which are related to diseases of the central nervous system [15,16,25]

The results indicate that extracts and compounds from this medicinal plant can modify behavioral responses induced with HAL, a non-selective agonist of dopaminergic D2 receptors, and with KET, a glutamatergic antagonist. Both neurotransmission systems are involved in psychotic symptoms, such as positive-negative-cognitive ones.

These psychotic behaviors share similar symptomatologic characteristics with diseases of cultural order, such as “madness” (dementia), which is reported as a set of traumatic symptoms that originate from a strong and sudden impression, which can threaten the physical and emotional integrity of the patient, who manifests sadness, nervousness, inability to communicate, with undesirable behaviors such as yelling, whistling, singing and muttering incoherencies, which lead to social rejection, forcing the individual to wander and sleep anywhere. In addition, it is mentioned that some types of “madness” cause the patient to present aggressive and hallucinatory states [10].

*T. lucida*, with the reports of the antidepressant, anxiolytic, and sedative effects in mice [16,26], is the basis of the present investigation, whose aim was to evaluate the possible antipsychotic effect of this plant. The first treatment for psychosis is the prescription of typical antipsychotics, whose mechanism of action focuses on antagonizing dopaminergic transmission mainly through D2 receptors. Furthermore, although they are effective in reducing the positive symptoms, it is also true that they do not counteract the negative and cognitive ones, and they also cause extrapyramidal effects. The first treatment for psychosis is the prescription of typical antipsychotics, whose mechanism of action antagonizes dopaminergic transmission mainly through D2 receptors. Moreover, although they are effective in reducing the positive symptoms, it is also true that they do not counteract the negative and/or cognitive ones, and they also cause extrapyramidal effects [27]. The cataleptic effect of HAL was used to define whether *T. lucida* interacts with the dopaminergic system by enhancing such activity. The decrement of DA transmission at postsynaptic D2 receptors has been implicated in catalepsy produced by antipsychotic drugs. Disrupting DA transmission with low doses of a D2 receptor-preferring antagonist such as haloperidol [28]. This drug is a potent and representative antipsychotic substance with a high affinity for dopaminergic D2 receptors. It is widely used in the laboratory to induce extrapyramidal effects in rodents, including stiffness, and particularly cataleptic effects that are mediated by the blockade of D2 receptors of the striatum. Catalepsy is evaluated when animals are placed on a raised bar on their front legs, and the time to the first movement is measured [29]. While the drug used as a control, caffeine, is an adenosine A2 receptor agonist which modulates the dopaminergic pathway blocking the catalepsy produced by HAL [30].

The extracts and compounds of *T. lucida* exert effects that can be classified as bi-phasic. In the VEH-Tween group, it was observed that catalepsy (considered as such when the forced and immobile posture of the mouse lasts more than 20 s) begins at time 60 and extends even at time 90. TlEA at 50 mg/kg potentiates catalepsy, but only in time 30 because after that, there are no changes compared with the VEH-Tween. However, in a 25 mg/kg dose, this variable is blocked similarly to caffeine. 

While treatment with TlAq (25 mg/kg) enhances it throughout the time course and up to 120 min, the effect starts from time 30; but again, a higher dose of this antagonized HAL. For the less polar extract, TlHex, none of the doses can enhance the cataleptic effect of the dopaminergic drug; beyond that, the 25 mg/kg dose blocks it, an action that decreases at a higher dose. Based on this behavior, which could be dose-dependent, it is then likely that catalepsy could be observed above 50 mg/kg. Something similar happens with TlMet, in which both doses block catalepsy, with a slight tendency for the high dose to have a lower capacity.

It is known that this plant has a significant secondary metabolism for the production of coumarins, which are widely described for this species. Coumarins are derivatives of 2H-1-benzopyran-2-one, widely distributed in nature; they are essential for their actions on different pharmacological systems since they have diverse activities, among which CNS disease models stand out. It is proposed that both natural and synthetic coumarins can be used to treat neurologic and neurodegenerative diseases. Such as Alzheimer’s and Parkinson’s disease, schizophrenia, epilepsy, nootropics, anxiolytics, or antidepressants, because they act on the neurotransmission systems involved in these diseases and are excellent anti-inflammatories [31]. In the present work and based on the results described by the research group [13], the isolation of DF and HN was achieved; they were evaluated at two different doses to investigate whether the effects of the extracts could fall on these substances.

For the catalepsy test, it was observed that DF at a dose of 1 mg/kg has the most significant potentiation effect on HAL, causing catalepsy. This effect appears from time 30, with a longer duration that lasts until time 120. At the same time, the 5 mg/kg dose has a lower activity level since the readings at times 60 and 90 are similar to the VEH group. Although catalepsy (considered for duration above 20 s) is no longer observed, the forced posture on the bar is longer than the VEH. The administration of herniarin provoked a biphasic effect; a low dose of 1 mg/kg is capable of potentiating HAL, increasing the onset, intensity, and time of catalepsy; but when the dose that is administered is 5 mg/kg, this coumarin now does completely block catalepsy, as caffeine does.

The difference between the effects caused by both coumarins may be based on small structural changes. For example, it can be seen that 1 mg/kg of the two coumarins potentiated the cataleptic effect of HAL. However, DF that is tri-methoxylated in positions 6, 7, and 8 exerts a more significant effect with a longer duration. When the dose was 5 mg/kg, a blockade of this activity was observed for herniarin, which has a methoxyl group in position 7. While the more complex structure (dimethylfraxetin) no longer presents a statistical difference with the VEH group throughout the time course and up to minute 90, the mice remain motionless on the bar longer. It is then observed that, at higher doses, the enhancing effect of catalepsy is lost and that the greater the chemical structure of coumarin, the behavior observed is lower.

With these considerations, we propose that extracts and coumarins of *T. lucida*, can interact with the receptors type D2 on which the HAL does so. That pharmacological association depends on the dose and chemical structure. Furthermore, although there are no reports in the literature of the effects of dimethylfraxetin (with three methoxyl groups in the coumarin structure at positions 6, 7, and 8) and herniarin (containing in its base structure a methoxy group at position 7) on the dopaminergic system, it is mentioned that other compounds of the same family modify this neurotransmission system. For example, scoparone, which has in its basic structure two methoxyl groups in positions 6 and 7, and that is also isolated from *T. lucida*, and whose anti-inflammatory activity has been described [13], is capable of inducing dopamine synthesis in PC12 cell cultures, as well as protecting them from L-3,4-dihydroxyphenylalanine (L-DOPA)-induced cytotoxicity [32].

Isosibiricin is coumarin, which has a keto group in position 8 of its structure instead of a methoxy, and which was isolated from the species *Murraya exotica* (Rutaceae). It increases the expression of dopaminergic D1/2 receptors in BV-2 microglia cell culture when stimulated with LPS through signaling mediated by NLRP3/caspase-1. Therefore, this coumarin is proposed as a modulator of the immune response mediated by dopaminergic receptors in the central nervous system [33]. Then herniarin and dimethylfraxetin, whose chemical structure is similar to those mentioned above, can interact with the dopaminergic system by modulating the HAL actions. In addition, the possibility that these coumarins may have a role in regulating other neurotransmission systems associated with psychoses is not ruled out.

Therefore, the next phase of the experiment consisted of evaluating the probable interaction of the products of this plant on the glutamatergic system. Since it has been demonstrated, for many years, that the classic theory of psychosis, based on dopamine, is only part of the constellation of neuronal intercommunication that leads to the triad of symptoms. The glutamate theory plays a fundamental role, specifically in the hypoactivity of the N-methyl-D-aspartate (rNMDA) receptor in the prefrontal cortex, which can cause psychosis [34]. So, to evaluate the antipsychotic effect of *T. lucida* and its possible interaction with rNMDA, KET was selected, a drug that acts mainly as an uncompetitive antagonist use-dependent of these receptors, among other targets on the central nervous system (CNS). It is valuable as a pharmacological tool to have an approach to the mode of action of treatments for schizophrenia. KET, widely used in psychiatry, has psychomimetic effects in humans and increases psychotic symptoms, such as dissociative states, altered perception, and schizophrenia-like positive and negative symptoms, in hospitalized patients [35]. This drug is used in many biological models, under different administration schemes, to reproduce some psychotic behaviors in rodents and mainly to identify possible treatments. The variability of pharmacological activities and even undesirable side effects that ketamine induces depend on factors such as the strain and species of rodent, the dose, the route of administration, and the dosage timing [36]. In the present work, and based Santillán-Urquiza et al. [37], the intraperitoneal route of administration was used in the scheme of a single dose of 50 mg/kg, which caused different behaviors in mice.

A passive avoidance test (PAT) was used to assess fear memory; this test is a valuable tool in searching for medication options for psychosis, especially in controlling cognitive symptoms. The present research observed that the administration of KET at 50 mg/kg causes cognitive deterioration in mice. This action was counteracted by N-methyl-D-aspartate and by extracts TlHex, TlEA, TlAq, herniarin, and dimethylfraxetin, isolated from *T. lucida*.

To measure the effectiveness of the treatments of this medicinal species on the negative symptoms of schizophrenia, the forced swimming test (FST) was widely used to evaluate the efficacy of synthetic drugs of natural origin. In this test, what is evaluated is the learned hopelessness; the animals tend to escape from the water, and thus the time of immobility is a behavioral marker of depression. The results here showed that KET decreases the immobility time of mice in the water cylinder, indicating an antidepressant effect of the drug, which is widely described. It was observed that the co-administration of treatments from *T. lucida* with KET does not modify its response, noting that TlMet diminished the effect of KET no-significatively, and only its antagonist, NMDA, was able to block the antidepressant effect.

The non-sociability of animals is used as a model to analyze possible pharmacological treatments to reduce unique negative symptoms in humans, so the FST and social interaction test (three-chamber social test) are helpful. This last trial focuses on the degree of familiarity between two individuals, and it has been observed that KET causes a decrease in the social interaction of rodents [38]. In this series of trials, which made it possible to measure cognitive deficit and negative symptoms, it was observed that the methanolic extract behaves differently from the other treatments derived from *T. lucida*, as it does not cause significant changes compared with the Veh-Tween group.

TlEA and TlAq, TlHex, interacted with KET in the memory and social interaction tests because they blocked this glutamatergic drug’s effect on the animals’ behavior in both tests. However, in the FST test, none of these treatments cause changes when compared with KET (Veh-Tween). These results indicate that *T. lucida*, in addition to interacting with the dopaminergic system, also exerts pharmacological effects on glutamate. By improving the condition of experimental psychosis, reducing memory deficits, and increasing social interaction. These actions may be attributable to their coumarin content; in this sense, herniarin, isolated from TlAq, and dimethylfraxetin, from TlEA, had similar pharmacological behavior to the extracts from which they were obtained. 

*T. lucida* also decreases hyperlocomotion and stereotyped behaviors observed in the OFT. Events that are attributed to the antagonism of NMDA receptors in mesolimbic GABAergic neurons, causing an increase in the excitability of these cells, and together with the ability of this substance to behave as a dopaminergic receptor agonist, it is an accessible model for the evaluation [39]. In the present work, it was possible to observe that the NMDA drug can block all these psychotic behaviors in mice. Furthermore, TlEA and TlAq extracts had the best effect since they block all the psychotic behaviors measured in the OFT (hyperlocomotion, the increase in rearings and groomings, time the mice spent grooming, the presence of stereotyped behaviors, and the time it took for them). Dimethylfraxetin and herniarin eliminate stereotypes and reduce the number of other behaviors.

## 4. Materials and Methods

### 4.1. Plant and Preparation of the Extracts

The aerial parts of *Tagetes lucida* Cav., were collected in 2018 in Xochitepec, Morelos, México. Margarita Aviles and Macrina Fuentes performed taxonomic identification. A voucher sample specimen was deposited at the Herbarium of the Instituto Nacional de Antropología e Historia Morelos (INAHM) and placed in the Medicinal Botanical Garden in Cuernavaca, Morelos, Mexico, with the code number INAHM-2086. The material was dried under dark conditions in a room-temperature ventilated place for two weeks. Afterward, to obtain particles 4–6 mm in size, the plant material was grounded in an electric grinder mill. The grounded material (4 kg) was placed in a glass flask for its first extraction with n-hexane for 24 h, and this procedure was carried out in triplicate. Then, the liquid extract was filtered and concentrated by distillation under reduced pressure in a rotary evaporator at 40 °C to obtain the hexane extract (TlHex). The residual plant material, once dry, was placed in a flask and was carried out in the same process to obtain the ethyl acetate (TlEA), methanol (TlMet), and aqueous (TlAq) extracts.

### 4.2. Isolation and Identification of Dimethylfraxetin (DF) and Herniarin (HN)

After the biological evaluation, extracts with the highest bioactivity were submitted to a chemical separation procedure for obtaining active compounds. TIEA and TlAq extracts were fractionated by open-column chromatography and carried out with successive columns of normal phase and reverse phase.

The TlEA extract (20 g) was separated following the described methodology by Porras Davila et al. [40] to obtain a fraction containing the dimethylfraxetin coumarin (DF). TlAq extract (277 g) was separated by chromatography in a column according to what is described to give herniarin (HN) [40].

The isolated compounds were selected for further evaluation in the biological model, identified as HN and DF, using HPLC (Waters, Milford, MA, USA) [13].

### 4.3. HPLC Analysis

The qualitative and quantitative analyses of dimethylfraxetin and herniarin were performed using an Alliance 2695 separation module system (Waters, Milford, MA, USA) coupled with a Spectral System UV2996 PDA detector. Chromatographic separation was carried out using a SupelcosilTM LC-F column (4.6 mm × 250 mm, 5 μm, Sigma-Aldrich, Bellefonte, PA, USA). The mobile phase consisted of two solvent reservoirs: A (Trifluoroacetic acid–water, 0.5%, *v*/*v*) and B (acetonitrile). The gradient system was as follows: 0–1 min, 100–0% B; 2–3 min, 95–5% B; 4–20 min, 70–30% B; 21–23 min, 50–50%, 24–25 min, 20–80% B, 26–27 min, 0–100% B and 28–30 min 100–0% B. The flow rate was 0.9 mL∙min^−1^ with an injection volume of 10 μL. The photodiode array detector wavelength was set at 325 and 280 nm to identify and quantify DF and HN, respectively. Calibration curves for these compounds were prepared by injecting ascendant concentrations of previously isolated compounds (Herniarin-HN-: 6.25, 12.5, 25, 50, and 100 μg∙mL^−1^; dimethylfraxetin-DF-: 40, 20, 10, 5, 2.5 and 1.75 μg∙mL^−1^). Analytical parameters of linearity, limit of detection and limit of quantification were measured dimethylfraxetin (*y* = 23493*x* + 382.5, R2 = 0.9995). for herniarin (*y* = 1575*x* − 86, R2 = 0.9971).

### 4.4. Treatments

The compounds, extracts, and substances utilized here, were administered orally (per os, p.o.) or intraperitoneally (i.p.) and included the following: TlHex, TlEA, TlMet, and TlAq extracts (25 and 50 mg/kg, p.o.); dimethylfraxetin, and herniarin (DF, HN 1 and 5 mg/kg, p.o.); haloperidol (HAL as catalepsy inducer 0.5 mg/kg, i.p.); caffeine (CAF, used as a positive control, to block catalepsy, 15 mg/kg, p.o.); vehicle (VEH, Tween 20, 1%, negative control, p.o.); ketamine (KET, schizophrenia-like symptoms inducer, 50 mg/kg, i.p.), and N-methyl-D-aspartate (NMDA, used as antagonist of KET, 50 mg/kg, i.p.). All drugs were purchased from Sigma-Aldrich, Co (St. Louis, MI, USA).

### 4.5. Animals

Experiments were performed on male ICR mice weighing between 30 and 35 g under controlled conditions in a light/dark cycle (12/12 h), at a temperature of 20 ± 2 °C, with free access to a special diet for rodents (Labdiet) and purified water. Prior to the test, animals were randomly divided into groups of 5. The research was conducted following the internationally accepted principles for laboratory animal use and care found in the National Institutes of Health (NIH) guidelines. The assays were developed in a soundproofed room with a video recording system; the experimenter avoided using a scent and remained silent. The experiments were performed according to official Mexican Norm 062-ZOO-1999 (Technical Specifications for the Production, Care, and Use of Laboratory Animals) and the international ethical guidelines for the care and use of laboratory animals. The experimental protocol was authorized by the local Health Research Committee [Mexican Institute of Social Security IMSS], with approval number: F-2019-1702-005.

### 4.6. Pharmacologic Tests

#### 4.6.1. Haloperidol-Induced Catalepsy

Catalepsy is observed when animals are placed in abnormal or unusual postures and maintain these postures for some time. For example, a typical animal will correct its position within seconds and explore its environment, but a cataleptic animal will maintain this externally imposed posture for a prolonged period.

Before catalepsy induction, mice were fasted for 1 h before the test and then administered the treatments derived from *T. lucida*. After 2 h, all groups received HAL, 0.5 mg/kg, i.p., and 10 minutes later, the test was started, and the cataleptic effect was evaluated at different time points (10, 30, 60, 90, 120, and 150 min). To measure the catalepsy, we employed the bar test (BT). The mice were placed for 1 min on a round glass bar (0.8 mm diameter) sustained by two wooden supports at a height of 4 cm and separated from 2.5-high horizontal support situated 2 cm from the middle portion of the glass bar. A positive response of catalepsy was considered when the mouse remained in a rigid position for 20 s or longer [37]. CAF (15 mg/kg, i.p.) was used as a standard drug in the catalepsy test. Therefore, it is expected that both the extracts and the isolated compounds can act potentiating the effects of haloperidol (cataleptic state), acting in this way through dopaminergic specifically via D2 receptors and not through A2 receptors as does the positive control group caffeine, considering that both heteroreceptors in a regulated manner diminish the excess of dopamine.

#### 4.6.2. Ketamine-Induced Psychosis

In order to study symptoms related to psychosis, mice were administered KET, which is an rNMDA antagonist. The study design lasted three days; on the first day, all treatments were administered; 24 h later, KET (50 mg/ kg, i.p.) and treatments were administered, lastly, on the third-day test was carried out, and treatments were administered for the third occasion 1 h before that started the test, all treatments (*T. lucida* derivatives) and NMDA were administered as described above. On test day, different behavioral tests were used, as described below.

#### 4.6.3. Passive Avoidance Test (PAT)

This unidirectional assay allowed us to study acquired learning and memory. The animal was conditioned with an aversive stimulus, which is later evaluated if the mouse remembers the experience. The assay consisted of placing the mouse in the lighted compartment, allowing it to explore for 1 min, and permitting it to direct itself to the dark compartment (typically, the mouse spontaneously tends to move into dark spaces, in that direct light produces anxiety in it). The time the mouse delays getting into the dark compartment (Initial Latency, IL) is measured; once the mouse is inside, the door is closed, and the mouse receives an electric discharge (0.6 mA, during 2 s). After application of the discharge, the mouse is returned to its cage until the time of evaluation, which was carried out 24 h later to evaluate long-term memory. On the test day, the animals were placed in the lighted camera, and the door that separates the two compartments stayed open. The electric discharge is not applied at this stage, the mouse is observed for 8 min, and the time is measured that the mouse delays in entering the dark compartment; Retention Latency (RL) is an indicator of memory [37].

#### 4.6.4. Forced Swimming Test (FST)

FST is a widely used model to evaluate the antidepressant effect, which is a characteristic symptom of schizophrenia induced by KET (Takeda et al., 2002) [41]. In this test, a previous training session that consisted of placing the mice individually for 15 min in a glass recipient with water, with a depth of 15 cm, at a temperature of 25 ± 2 °C, was carried out. To ascertain whether the mouse identified that there was no way for it to escape. Then, mice were placed in the glass recipient containing water for 5 min. Total immobility time was measured for each mouse [37].

#### 4.6.5. Open Field Test (OFT)

This test is used to measure the positive symptoms of schizophrenia. In this model, the reactions of rodents are observed in an environment without escape (Walsh and Cummins, 1976) [42]. It is currently used to study motor activity and allows us to analyze the excitatory or depressive effect of the locomotor system. Mice were placed on the OFT platform, and their behavior was videotaped by a digital camera fixed above the field. The observation was maintained for 30 min, and we measured the following parameters of locomotion as Total Crossings (TC); Rearing (R); Stereotyped Behaviors (SB), which are repetitive, involuntary movements with no apparent purpose, such as head-spinning, Time Spend on Stereotyped Behaviors (TSB); Grooming (G), and Time Spend on Grooming (TSG) [37].

#### 4.6.6. Social Interaction Test (SIT)

This test focuses on the degree of familiarity of two individuals, allowing us to observe rodents’ behavior on negative and cognitive symptoms of schizophrenia. It consists of a compartment divided into three spaces, 2 of which on the sides contain circular chambers. The test begins with habituation, placing the mouse in the main cage for 10 minutes to become familiar with the environment and leaving the circular chamber empty. Subsequently, in one of the compartments of the circular chamber, a mouse is placed that we will call “family” since it was previously in contact for five minutes with the experimental mouse. A new mouse is placed in the other compartment, which has never been exposed to the experimental mouse. The experience is performed four times in five-minute sessions. First, the animal is confronted with a familiar mouse, and during these sessions, the mouse gets used to this exposure and varies its social investigation (habituation). In the fourth exposure, the camera is rotated, and the new mouse is shown (dishabituation). The exploration time of the fourth day is recorded, defined as the time that the animal spends approaching or sniffing the mating window. Mice with unimpaired cognitive function will spend more time sniffing the new mouse than the familiar one; those with a cognitive deficit will not distinguish between the familiar mouse and the new one (their exploration time will be similar) [29].

### 4.7. Statistical Analysis

The data were expressed as the means ± standard error of the mean (S.E.M.), and statistical significance was determined using an analysis of variance (ANOVA) followed by Dunnett’s test compared with the tween group (Vehicle, Veh-Tween). Values considered significant at *p* < 0.05 was employed to define significant differences among the groups. SPSS version number 11.0 (IBM SPSS, New York, NY, USA) software was used for graphing and statistical analysis.

## 5. Conclusions

The pharmacological activity of *Tagetes lucida* extracts can be attributed in part to the presence of coumarin-type compounds, such as HN and DF, which have activity as potentiators of catalepsy and blockers of psychotic observed behaviors, indicating that the plant and Some of its components are capable of interacting with the dopaminergic and glutamatergic systems, both important mechanisms involved in psychosis and they can be attributed to this type of compounds as responsible for the activity of *Tagetes lucida* extracts. These coumarins behave pharmacologically similar to the extracts, so they have attributed activity. It is shown for the first time that *T. lucida* interacts with the dopaminergic and glutamatergic systems, both important mechanisms involved in psychosis.

## Figures and Tables

**Table 1:** Effect of *T. lucida* extracts, herniarin (HN), and dimethylfraxetin (DF) on acute schizophrenia-like symptoms induced with KET 50 mg/kg in the Open Field Test (OFT) in mice. (Observation time 30 min).

Treatments (mg/kg)	TC (Events)	R (Events)	G (Events)	TSG (sec)	SB (Events)	TSB (sec)
Baseline	235.0 ± 14.6 *	135.2 ± 11.4 *	27.8 ± 0.7 *	185.4 ± 9.7 *	0.0 ± 0.0 *	0.0 ± 0.0 *
**KET (50 mg/kg)**						
VEH-Tween	435.6 ± 9.2	225.0 ± 15.3	42.2 ± 3.7	272.6 ± 8.0	13.8 ± 1.2	37.6 ± 5.5
NMDA 5	267.6 ± 14.9 *	71.6 ± 2.6 *	20.4 ± 3.6 *	190.0 ± 29.3 *	0.0 ± 0.0 *	0.0 ± 0.0 *
TlHex 50	227.8 ± 23.3 *	144.2 ± 18.4 *	22.8 ± 2.8 *	223.4 ± 31.9	1.0 ± 0.6 *	33.2 ± 19.2
TlEA 50	216.4 ± 30.8 *	95.2 ± 17.7 *	24.4 ± 4.0 *	176.4 ± 7.8 *	0.0 ± 0.0 *	0.0 ± 0.0 *
TlMet 50	238.6 ± 6.0 *	240.8 ± 11.3	49.2 ± 5.6	194.0 ± 3.4 *	4.0± 1.76 *	5.8 ± 1.5 *
TLAq 25	209.6 ± 34.9 *	124.8 ± 12.9 *	17.2 ± 2.2 *	105.0 ± 22.1 *	0.4 ± 0.4 *	2.0 ± 2.0 *
HN 1	169.6 ± 12.9 *	63.2 ± 7.6 *	27.2 ± 2.8 *	342.0 ± 75.2	0.0 ± 0.0 *	0.0 ± 0.0 *
DF 1	246.6 ± 23.7 *	132.2 ± 12.8 *	20.2 ± 1.7 *	187.6 ± 47.6 *	0.0 ± 0.0 *	0.0 ± 0.0 *

Total crossings (TC), rearings (R), groomings (G), time spent grooming (TSG), stereotyped behaviors (SB), and time spent stereotyped behaviors (TSB). ANOVA followed by Dunnett’s Test ($\bar{x}$ ± S.E.M., n = 5) with * *p* < 0.05 when the comparison is with the VEH-Tween group.

## Data Availability

Not applicable.
